# The impact of parental posttraumatic stress disorder on parenting: a systematic review

**DOI:** 10.1080/20008198.2018.1550345

**Published:** 2019-01-14

**Authors:** Hope Christie, Catherine Hamilton-Giachritsis, Filipa Alves-Costa, Mark Tomlinson, Sarah L. Halligan

**Affiliations:** a Department of Psychology, University of Bath, Bath, UK; b Department of Forensic and Neurodevelopmental Sciences, Kings College London, London, UK; c Department of Psychology, Stellenbosch University, Stellenbosch, South Africa

**Keywords:** Posttraumatic stress disorder, trauma, parenting, parent–child relationship, family, Trastorno de estrés postraumático, trauma, crianza, relación padre-hijo, familia, 创伤后应激障碍, 创伤, 教养, 亲子关系, 家庭

## Abstract

**Background**: Posttraumatic stress disorder (PTSD) is a serious and debilitating disorder that can develop following exposure to a traumatic event. Where parents develop PTSD, it may have an impact on their parenting role.

**Objective**: The objective was to review the existing evidence base on parental PTSD, examining whether parental PTSD has an impact on key parenting domains.

**Method**: A comprehensive web-based search identified 27 quantitative studies that examined parental PTSD in relation to parenting domains.

**Results**: Several parenting domains were investigated including: parenting satisfaction, parenting stress, the parent–child relationship, and specific parenting practices. Sample sizes ranged from 19 to 3931 parents. A range of parental traumas were investigated, including traumatic birth experiences, military trauma, and intimate partner violence. Findings indicated associations between parental PTSD and several domains of parenting, but there were inconsistencies across studies.

**Conclusions**: Findings suggested that parental PTSD is associated with impaired functioning across a number of parenting domains, including increased levels of parenting stress, lower parenting satisfaction, less optimal parent–child relationships, and more frequent use of negative parenting practices, such as overt hostility and controlling behaviours. However, methodological limitations across the literature as a whole limited the potential to infer causal impacts of PTSD on parenting. Further study is also needed to advance our current understanding around the impact of different trauma types on parenting domains.

## HIGHLIGHTS

• The review brought together existing evidence in the parental PTSD literature, to further understand how parental PTSD impacts parenting domains.• A total of 27 articles were reviewed, examining several parenting domains including: parenting satisfaction, parenting stress, the parent–child relationship, and specific parenting practices.• Parents with PTSD may experience more impaired functioning in these specific domains compared to parents without PTSD.• Methodological limitations across the literature limit potential inference from the current findings; therefore further study is still needed.

## Abstract (es)

**Antecedentes**: El trastorno de estrés postraumático (TEPT) es un trastorno grave y debilitante que puede desarrollarse después de la exposición a un evento traumático. El hecho que los padres desarrollen TEPT, puede tener un impacto en su rol parental de crianza.

**Objetivo**: El objetivo de esta revisión fue revisar la base de evidencia existente sobre el TEPT de los padres, y analizar si el TEPT de los padres tiene un impacto en los dominios de crianza de los hijos.

**Método**: una búsqueda exhaustiva a través de internet identificó 27 estudios cuantitativos que examinaron el TEPT de los padres en relación con los dominios de crianza.

**Resultados**: se investigaron varios dominios de crianza de los hijos, incluidos: satisfacción parental, estrés parental, la relación padre-hijo y prácticas de crianza específicas. Los tamaños de muestra oscilaron entre 19 y 3931 padres. Se investigó una variedad de traumas parentales, que incluyeron experiencias traumáticas de nacimiento, traumas militares y violencia de pareja. Los hallazgos indicaron asociaciones entre el TEPT de los padres y varios dominios de crianza de los hijos, pero hubo inconsistencias entre los estudios.

**Conclusiones**: los hallazgos sugirieron que el trastorno de estrés postraumático de los padres se asocia con un funcionamiento deficiente en varios dominios de crianza de los hijos, incluido un mayor nivel de estrés parental, menor satisfacción parental, relaciones menos óptimas entre padres e hijos, y un uso más frecuente de prácticas de crianza negativas, como la hostilidad manifiesta y conductas controladoras. Sin embargo, las limitaciones metodológicas en toda la literatura en su conjunto limitaron el potencial para inferir los impactos causales del TEPT en la crianza de los hijos. Aún se necesita más estudios para avanzar en nuestra comprensión actual sobre el impacto de los diferentes tipos de trauma en los dominios de crianza.

## Abstract (zh)

**背景**：创伤后应激障碍（PTSD）是一种带来严重损耗的疾病，可在创伤事件后发生。如果父母患有创伤后应激障碍，可能会影响他们的教养角色。

**目的**：本文的目的是综述现有的父母创伤后应激障碍的证据基础，考察父母创伤后应激障碍是否对关键的教养领域产生影响。

**方法**：在网络上进行综合搜索确定了27项定量研究，这些研究考察了父母PTSD与教养的关系。

**结果**：调查了几个教养领域，包括：教养满意度，教养压力，亲子关系和特定教养实践。样本量范围从19到3931名父母，考察了一系列父母创伤经历，包括创伤性出生经历、军事创伤和亲密伴侣暴力。研究结果显示，父母PTSD与教养的几个领域之间存在关联，但各研究之间存在不一致。

**结论**：研究结果表明，父母创伤后应激障碍与许多教养领域的功能受损相关，包括教养压力水平升高，教养满意度降低，亲子关系不理想，以及更频繁地使用消极教养方式，如明显的敌意和控制行为。然而，整个文献中的方法学局限性限制了推断创伤后应激障碍对教养的因果影响的可能性。以后还需要进一步研究，以推进我们对不同创伤类型对教养领域的影响的理解。

**Abbreviation**

DSM: Diagnostic Statistical Manual for Mental Disorders

## 1. Introduction

Posttraumatic stress disorder (PTSD) may develop following a traumatic event, and is estimated to have a lifetime prevalence of 7.8% (Kessler, Sonnega, Bromet, Hughes, & Nelson, 1995). A considerable number of adults who develop PTSD are also parents with dependent children (Lauterbach et al., 2007; Leen-Feldner, Feldner, Bunaciu, & Blumenthal, 2011). Psychological difficulties in adults may impair parenting capabilities. PTSD in particular can cause negative alterations to an individual’s behaviour, including increased anger and reactivity, as well as social withdrawal (American Psychiatric Association, 2013). Further, as highlighted by a wealth of research, trauma has the potential to impact across generations, which may also cause subsequent psychological, social, and emotional difficulties in children (Berg-Nielsen, Vikan, & Dahl, 2002; van Ee, Kleber, & Jongmans, 2016). As such, it is essential to understand the potential consequences of PTSD for parental functioning. Recent syntheses of relevant aspects of the literature have considered the potential detrimental role of parental PTSD from several standpoints, including in refugee families (van Ee et al., 2016), for military veterans and their families (Creech & Misca, 2017), and in relation to children’s outcomes in the context of parental PTSD (Lambert, Holzer, & Hasbun, 2014; Leen-Feldner et al., 2013; Morris, Gabert-Quillen, & Delahanty, 2012). However, there remains a lack of a comprehensive and critical synthesis of the parental PTSD literature relating to possible impacts on parenting domains that cuts across trauma populations. An understanding of whether impacts of parental PTSD are present that generalize across different trauma types is thereby limited.

**Table 1.** Summary of studies included in review.

Reference(study type)	Sample size	Age years (*M*, range)	Comparator group(s)	Parenting outcome	Measures	Trauma type	Quality score (%)
1. Ayers et al. 2007 (Cross-sectional)	64 couples	32.4	Severe PTSD symptoms *n =* 4 couples	Bonding	Adapted IES, BMIIS	Birth	47
2. Berz et al. 2008 (Cross-sectional)	60 mothers	49.1	Correlational study	Satisfaction	M-PTSD, Parenting Satisfaction Scale	Military	63
3. Bosquet Enlow et al. 2014 (Cross-sectional)	45 dyads (mothers)	27.04	Elevated PTSD symptoms *n =* 12; Non-elevated PTSD symptoms *n =* 33	Attachment	PCL-C, SSP	Various, unspecified	97
4. Chemtob & Carlson, 2004 (Cross-sectional)	25 dyads (mothers)	35.4	PTSD group *n =* 11; No PTSD group = 13	Inconsistent discipline	PTDS, Parenting Scale	IPV	84
5. Chemtob et al., 2013 (Cross-sectional)	97 dyads (mothers)	22–30	Correlational study	Parenting stress	PDS, PSI-SF	Various, unspecified	75
6. Cohen et al. 2011 (Cross-sectional)	477 fathers	46.9 CSR group; 47.59 non-CSR group	PTSD group *n =* 124; No PTSD group *n =* 353	Satisfaction	PTSD Inventory, PFQ	Military	88
7. Creech et al. 2017 (Cross-sectional)	134 mothers	37.11	Correlational study	Satisfaction	PCL, Parenting Sense of Competence Scale	Military	81
8. Cross et al. 2017 (Cross-sectional)	112 dyads (mothers)	Not provided	Correlational study	Child abuse potential	MPSS, CAPI	Various, unspecified	81
9. Davies et al. 2008 (Cross-sectional)	211 mothers	26.13 FS; 30.40 PS; 30.21 NS	Fully symptomatic (FS) *n =* 8; Partially symptomatic (PS) *n =* 45; Non-symptomatic (NS) *n =* 158	Overt hostility	SCID-PTSD, PTSDQ, IES, MORS-SF, MPAS	Birth	87
10. Forcada-Guex et al. 2011 (Cross-sectional)	72 dyads (mothers)	Not provided	Control group *n =* 25; Low PTSS *n =* 31; High PTSS *n =* 16	Attachment	PPQ, WMCI, 10 min Interactive Play Session	Birth	69
11. Gewirtz et al. 2010 (Longitudinal)	468 fathers	36.36	Correlational study	Child abuse potential	PCL-M, APQ-9	Military	75
12. Hershkowitz et al. 2017 (Longitudinal)	200 parents	37.20 (23–59)	Correlational study	Child abuse potential, Satisfaction	PDS, APQ-9, PSQ	Various, unspecified	78
13. Ionio & Di Blasio, 2014 (Cross-sectional)^1^	19 mothers	32.23	Clinical PTSD *n =* 4; No PTSD *n =* 15	Controlling behaviour	PPQ, SFP	Birth	66
14. Jobe-Shields et al. 2016 (Cross-sectional)	96 caregivers	Not provided	No distress *n =* 73; PTSD group *n =* 7; Depression group *n =* 10; PTSD and Depression *n =* 6	Inconsistent discipline	PSS-SR, APQ	Indirect	75
15. Jordan et al. 1992 (Cross-sectional)	1200 fathers	PTSD group = 39.8; Non-PTSD group = 41.81	PTSD group *n =* 319; No PTSD group *n =* 871	Satisfaction	M-PTSD, PPI	Military	66
16. Lauterbach et al. 2007 (Cross-sectional)	Unclear	Unclear	Unclear	Parent–child Relationship	DIS, PCRQ	Various, unspecified	59
17. Leen-Feldner et al. 2011 (Cross-sectional)	2228 mothers; 1703 fathers	50.02	PTSD group *n =* 286; No PTSD group *n =* 3645	Overt hostility	WHOCIDI, Parental Aggression Scale	Various, unspecified	87
18. Marsanic et al. 2014 (Cross-sectional)	244 dyads (fathers)	45.7	PTSD group *n =* 122; No PTSD group *n =* 122	Controlling behaviour	YSR, FAD, PBI	Military	71
19. Parfitt & Ayers, 2009 (Cross-sectional)	126 mothers; 56 fathers	Mothers 30.92; Fathers 32.58	PTSD group *n =* 31; No PTSD group *n =* 121	Parent–child Relationship	PDS, PBQ	Birth	66
20. Salloum et al., 2015 (Cross-sectional)	43 dyads	38.78 (24–73)	Correlational study	Parenting stress	SCID-RV, PSI-SF	Various, unspecified	87
21. Samper et al. 2004 (Cross-sectional)	205 fathers	41.44 (33–62)	Correlational study	Satisfaction	M-PTSD, Parenting Satisfaction Scale	Military	66
22. Schechter et al. 2015 (Cross-sectional)	56 mothers	34	PTSD group *n =* 34; No PTSD group *n =* 22	Controlling behaviour	CAPS, PCL-S, 5 min free play	IPV	72
23. Schechter et al. 2010 (Cross-sectional)	74 mothers	29.39	PTSD group *n =* 17; Subthreshold PTSD symptoms *n =* 30; No PTSD *n =* 27	Limited emotional availability	CAPS, PCL-S, AMBI, CJAS	IPV	69
24. Solomon et al. 2011 (Cross-sectional)	473 parents	46.9 CSR group; 47.59 non-CSR group	PTSD group *n =* 123; No PTSD group *n =* 350	Overall poor parenting	PTSD-I, Parental Functioning	Military	56
25. Suttora et al., 2014 (Cross-sectional)	243 mothers	34.2 Preterm group; 34.4 full-term group		Parenting stress	PPQ, PSI-SF	Birth	65
26. Vukovic et al. 2015 (Cross-sectional)	324 dyads (fathers)	Not provided	PTSD group *n =* 108; Partial PTSD group *n =* 108; No PTSD group *n =* 108	Controlling behaviour	YSR, PBI	Military	75
27. Wilson et al., 2017 (Cross-sectional)	52 mothers	34.77	Correlational study	Parenting stress	PCL-C, PSI-SF	Various unspecified	81

**Note. ^1^** Although the overall study design was longitudinal, analyses relevant to this review were wholly cross-sectional. AMBI = The Atypical Maternal Behaviour Instrument; APQ = Alabama Parenting Questionnaire; APQ-9; Alabama Parenting Questionnaire (short form); BMIIS = Bethlehem Mother–Infant Interaction Scale; CAPI = Child Abuse Potential Index; CAPS = Clinician Administered PTSD Scale; CJAS = Coordinated Joint Attention Scales; DIS = Diagnostic Interview Schedule; IES = Impact of Events Scale; MORS-SF = Mothers’ Object Relations Scale-Short Form; MPAS = Maternal Postnatal Attachment Scale; M-PTSD = Mississippi Combat Scale; PBI = Parental Bonding Instrument; PBQ = Postpartum Bonding Questionnaire; PCFS = Perceived Child Functioning Scale; PCL-C = Posttraumatic Stress Disorder Checklist – Civilian; PCL-M = Posttraumatic Stress Disorder Checklist – Military; PCL-S = Post-traumatic Symptom Checklist – Short Version; PCRQ = Parent–Child Relationship Quality; PDS = Posttraumatic Diagnostic Scale; PFQ = Parental Functioning Questionnaire; PPI = Parental Problems Index; PPQ = Perinatal Posttraumatic Stress Disorder Questionnaire; PSI-SF = Parenting Stress Index-Short Form; PSQ = Parenting Satisfaction Questionnaire; PSS-SR = Posttraumatic Stress Disorder Symptom Scale–Self Report; PTSD-I = Posttraumatic Stress Disorder Inventory; PTSDQ = Posttraumatic Stress Disorder Questionnaire; SCID-PTSD = Structured Clinical Interview for DSM-IV Axis I Disorders; SFP = Still Face Paradigm; SSP = Strange Situation Procedure; WHOCIDI = World Health Organization World Mental Health Composite International Diagnostic Interview; WMCI = Working Model of the Child Interview; YSR = Youth Self Report

**Figure 1.**
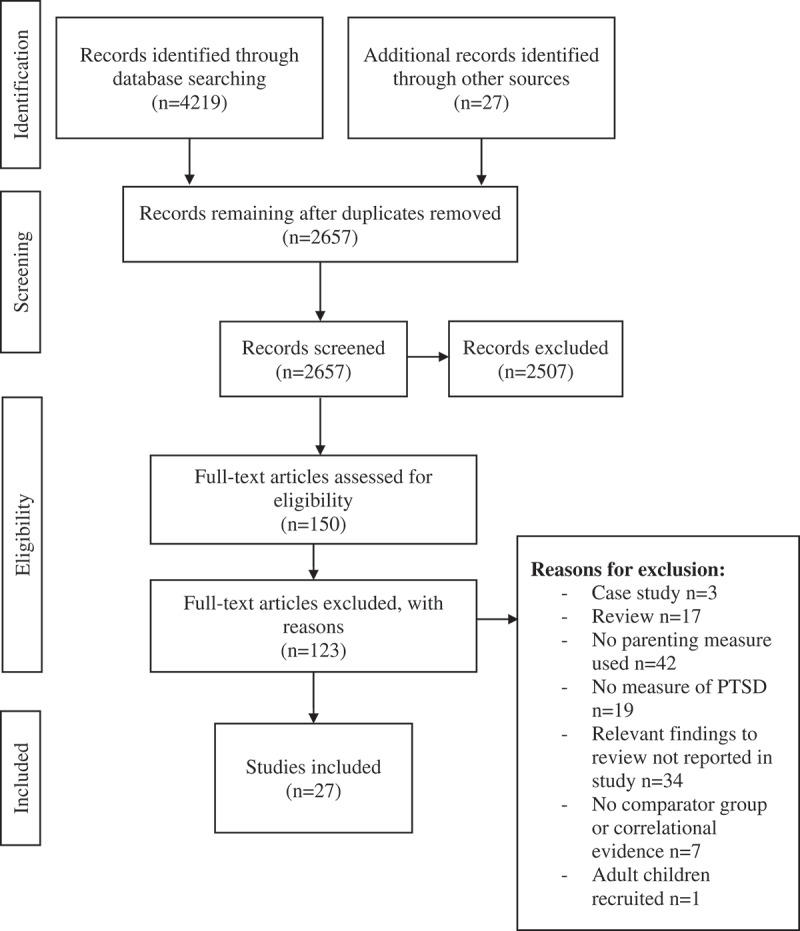
Flow chart of study selection process.

The aim of the current systematic review was to examine the evidence base in the field of parental PTSD in order to address the research question: what impact does parental PTSD have on parenting domains and the parent–child relationship? Given the relatively preliminary stage of literature in this area, a broad definition of parenting domains was applied (Berg-Nielsen et al., 2002; O’Connor, 2002). Thus, we included studies that indexed: the parent’s behaviour towards their child (e.g. warmth/support, hostility, overprotection); the quality of the parent–child relationship (including attachment styles and bonding impairments); and the parent’s thoughts and feelings about their own parenting ability (parenting satisfaction versus stress).

## 2. Method

A protocol for the review was published via PROSPERO (registration number: CRD42016040175).

### 2.1. Literature search strategy

Searches were conducted using PubMED, PsycInfo, PsycNet, and Published International Literature of Traumatic Stress (PILOTS) for articles published between 1980 (when PTSD was first introduced in the DSM) and December 2017.

The following search terms were used: ‘posttraumatic stress disorder*’ OR ‘post-traumatic stress disorder*’ OR ‘post traumatic stress disorder’ OR ‘PTSD’, AND ‘parent*’ OR ‘parental*’ OR ‘mother*’ OR ‘father*’ OR ‘maternal*’ OR ‘paternal*’ OR ‘caregiver*’. The search terms were broad in order to conduct a comprehensive search of the research field. In addition, reference lists of relevant review papers and book chapters were manually searched for articles that may not have been identified in the literature search. Four key authors were contacted to request any further published or unpublished studies that could potentially be included. A Preferred Reporting Items for Systematic Reviews and Meta-Analyses (Moher, Liberati, Tetzlaff, Altman, & Group, 2009) flowchart is provided in Figure 1.

The first author conducted the literature search, screened articles, and extracted data. Two authors (HC and FAC) discussed the inclusion and exclusion of 25 randomly selected papers, from any stage of the screening process, in order to assess reliability, consistency and academic rigour. Cohen’s kappa indicated a substantial level of agreement between raters (*k* = 0.684, *p* = .001). Any disagreements were discussed with two additional authors until a consensus was reached. Following a consensus agreement on inclusion or exclusion of articles, the first author then revisited previously excluded articles in order to ensure that no other articles previously excluded should now be included.

### 2.2. Eligibility criteria

Studies were included if they: (a) had used a measure of parenting; (b) used a validated measure of PTSD; and (c) included parents who had PTSD during their offspring’s childhood, with childhood defined as 0–18 years of age. Studies were excluded if they: (a) did not have *either* a comparator group that did not have PTSD *or* correlational evidence of associations between PTSD symptom severity and parenting outcome scores; (b) had recruited parents based on knowledge that they were abusing their children (as selection of sample based on serious parenting concerns would clearly introduce bias); (c) recruited parents on the basis of another disorder being present (i.e. PTSD was only studied when comorbid with another specific problem), as this precludes conclusions about potential for causal impact of PTSD in particular.

All included articles underwent a quality assessment using the Hawker’s Checklist (Hawker, Payne, Kerr, Hardey, & Powell, 2002), which is designed to limit bias that may be introduced while synthesizing evidence. The checklist provides a standardized list in order to score and rate the overall quality of papers based on nine categories, on a scale of 0 (poor quality) to 4 (good). We used eight of the nine categories, covering clarity (e.g. ‘was there a good background and clear statement of the aims of the research?’), quality of the results, and the generalizability and transferability of findings (e.g. ‘Has the context and setting been described sufficiently to allow comparison with other contexts and settings?’). Ratings relating to title and abstract clarity were excluded, as they were judged not to provide a relevant indication of quality in the current synthesis. Results of these assessments can be found alongside the study characteristics in the results section, presented as a proportion of the total possible score of 32 (see Hawker et al., 2002, for more information).

## 3. Results

### 3.1. Overview of reviewed studies

The 27 studies^1^ identified investigated parenting domains including: parenting satisfaction and stress, the parent–child relationship (both bonding and attachment), and a range of specific parenting practices (e.g. overprotection and hostility), each of which are discussed in detail below. All studies were quantitative, with sample sizes ranging from 19 to 3931. Parental traumas included birth experiences (*k* = 6 studies), military trauma (*k* = 9), intimate partner violence (IPV; *k* = 3), learning that their child had been maltreated (*k* = 1), and unspecified mixed traumas (*k* = 8). A comprehensive summary of each study can be found in Table 1.

### 3.2. Parenting satisfaction

Parenting satisfaction encompasses perceptions of parenting efficacy, and enjoyment gained from parenting (Cohen, Zerach, & Solomon, 2011). Six studies investigated the impact of PTSD on parenting satisfaction; all but one used military samples (Berz et al., 2008; Cohen et al., 2011; Creech et al., 2016; Hershkowitz et al., 2017; Jordan et al., 1992; Samper et al., 2004). Studies were rated moderate to high in terms of quality (range 20–28 out of 32, corresponding to .63–.88).

Jordan et al. (1992) found that veteran fathers with PTSD (*n =* 231) reported lower parenting satisfaction on a brief self-report measure compared to veteran fathers without PTSD (*n =* 736), although the effect size was small (Phi = 0.27). Similarly, Cohen et al. (2011) investigated the impact of paternal PTSD in a sample of 477 veterans. Fathers who suffered from PTSD (*n =* 124) self-rated their parenting satisfaction significantly lower than those who did not have PTSD (*n =* 353). Samper et al. (2004) reported similar findings with a sample of male veterans (*n =* 250) when investigating associations between PTSD symptom clusters and parenting satisfaction, using a five-item measure. Overall PTSD symptom scores (*r =* −0.27) and avoidance scores (*r =* −0.30) were significantly negatively associated with parenting satisfaction, with small to medium effect sizes, but there were no equivalent associations for hyperarousal or re-experiencing symptoms. Effects were retained controlling for factors including major depression, alcohol abuse, and intimate partner violence. Berz et al. (2008) studied a sample of female veterans (*n =* 60) using the same five-item parenting satisfaction measure and found avoidance (*r =* −0.23) and hyperarousal (*r =* −0.29), but not re-experiencing, to be inversely associated with satisfaction scores. However, contrary to previous findings, Creech et al. (2016) found no significant associations between military veteran mothers’ (*n =* 64) PTSD symptom scores and their parenting satisfaction scores.

While this body of work has been predominantly military focused, Hershkowitz et al. (2017) found that in a sample of trauma exposed parents from the general population (*N =* 200), PTSD symptoms showed an inverse association with parenting satisfaction (*r =* −0.46, indicating a medium effect), but this effect was eliminated once factors including depression, age, and number of children were included in the model. Overall, the small body of work in this area is consistent in suggesting that veteran fathers may experience reduced satisfaction in their parenting role due to the presence of PTSD, although this impact is small in magnitude. More work is needed to establish whether similar effects apply in other populations.

### 3.3. Parenting stress

Parenting stress can be defined as the ‘aversive psychological reaction to being a parent’ and is noted to potentially be related to parenting behaviours and child outcomes (Deater-Deckard, 1998, p. 315). We identified four studies that investigated the impact of parental PTSD on parenting stress following birth trauma (Suttora et al., 2014) and mixed traumas (including domestic violence, sexual abuse, physical abuse; Chemtob, Gudino, & Laraque, 2013; Salloum et al., 2015; Wilson et al., 2017). Parenting stress was consistently measured using the short form of the Parenting Stress Index (PSI-SF; Abidin, 1995). The mean quality rating for papers was 24.75 (range .65 to .87). In a small, heterogenous trauma sample of mother–child dyads (*N =* 43), Salloum et al. (2015) found no significant associations between PTSD symptom scores and the three PSI-SF subdomains of parental distress, parent–child dysfunction, or the parent’s perception of difficult behaviour from the child. By contrast, three studies that studied mothers using only the total PSI-SF score each found links with PTSD. Suttora et al. (2014) studied mothers who had given birth to a pre-term infant (*n =* 87) and mothers of full-term infants (*n =* 156) and found that PTSD symptoms mediated an association between birth status and total parenting stress. Wilson et al. (2017), in a mixed trauma sample of mother–child dyads (*N =* 52), found that PTSD symptoms were significantly associated with parenting stress score, with medium effect (*r* = 0.30). Lastly, in a community sample of mothers, a proportion of which reported trauma exposure, Chemtob et al. (2013) compared groups with no diagnosis (*n =* 70), PTSD-only (*n =* 6), depression only (*n =* 11), and co-morbid depression and PTSD (*n =* 10), based on questionnaire scores. All diagnostic groups reported elevated parenting stress, with no differences between them. Findings are limited by the small sample and the lack of a trauma control group. Further research is needed to tease apart the constructs measured by the PSI, which include child as well as parent and relationship characteristics, in order to provide more clarity in this area.

### 3.4. Parent–child bonding and relationships

Six studies focused on the impact of parental PTSD on the parent–child relationship, including measures of relationship quality and the mutual parent–child emotional bond, the attachment that the child develops for the parent, and the representation of the attachment held by the parent in relation to the child. Four studies utilized birth trauma samples (Ayers, Wright, & Wells, 2007; Davies, Slade, Wright, & Stewart, 2008; Forcada-Guex, Borghini, Pierrehumbert, Ansermet, & Muller-Nix, 2011; Parfitt & Ayers, 2009), and two used samples from cohort studies that had experienced various, unspecified traumas (Bosquet Enlow, Egeland, Carlson, Blood, & Wright, 2014; Lauterbach et al., 2007). Quality ratings for studies ranged from poor (.27) to excellent (.97).

Several studies focused on parental perceptions of their relationship with their child. An internet-based study of parents focused on negative birth experiences (*N =* 152; 126 women) found symptoms of PTSD to be correlated with more self-rated difficulties in the bond with their baby (*r* = .36; Parfitt & Ayers, 2009). When symptoms of depression were adjusted for in a structural equation model, a small independent effect of PTSD was retained (*d =* .20). Symptoms of PTSD were also associated with more problematic birth characteristics, but these were not accounted for in analyses. Similarly, mothers and fathers deriving from a cohort study who met the diagnostic criteria for PTSD related to mixed traumas (*n =* 323) were found to rate their relationship with their child as being significantly poorer than parents without PSTD (*n =* 5884) but the effect was very small (*n*^2^ = 0.005; Lauterbach et al., 2007). The validity of these findings is limited by the use of a single item measure of the parent–child relationship. Importantly, the study also lacked a trauma exposed control group. By contrast, Ayers et al. (2007) studied 64 couples post-birth and found no associations between PTSD symptoms in either mother or father and a poorer bond with their baby. However, overall symptoms were low in this study, which did not focus specifically on traumatic birth experiences. Further, the quality score for Ayers et al. (2007) was low due to this and other factors, including the measurement of only two of the four symptom clusters of PTSD.

Several studies in this area examined parental attachment to the child. In a study of maternal self-reported attachment perceptions, Davies et al. (2008) found that post-birth symptoms of PTSD in mothers (*N =* 211) were moderately associated with a perceived poorer quality of attachment to their infant at six-weeks postpartum. However, controlling for the effect of postnatal depression eliminated these effects in this sample, the majority of whom had experienced normal deliveries (Davies et al., 2008). Forcada-Guex et al. (2011) compared mothers of pre-term infants with low (*n* = 31) or high levels (*n* = 16) of PTSS to mothers of full-term infants (*n* = 25; i.e. a no trauma group). Overall, fewer mothers with pre-term versus full-term infants were classed as having balanced attachment representations, as measured by the Working Model of the Child Interview. Underpinning this, there was tentative evidence that relative to full-term mothers, mothers in the low PTSS group were more likely to have disengaged attachment representations, whereas those in the high PTSS group were more likely to have distorted attachment representations. However, no comparisons revealed significant differences between low and high PTSS groups, meaning that PTSS effects were not clearly demonstrated.

One study examined the attachment the infant formed to the parent (Bosquet Enlow et al., 2014). Mothers from low-income backgrounds, 80% of whom reported lifetime exposure to potentially traumatic events, were categorized as PTSD (*n* = 12) or no PTSD (*n* = 33) based on questionnaire scores at six-months postpartum. Compared to the no PTSD group, infants of mothers in the PTSD group were significantly less likely to have a secure attachment at six months (OR = 11.31, indicating a large effect size), and the likelihood of a disorganized attachment classification was particularly elevated (OR = 13.17). Presence of depressive symptoms and extent of trauma history did not appear to account for these effects. The use of the Strange Situation Paradigm (Ainsworth, 1969) to measure attachment was a major strength of this research, but larger scale studies of this type are needed to confirm the findings. Overall, these findings are mixed, suggesting that in some situations parents with PTSD’s relationship with their child may be perceived as poorer than those without PTSD. However, the majority of studies have examined this through self-report measures. While insightful, these measures may be influenced by the parent’s mental health. As such, more replication of observational studies is required.

### 3.5. Parenting practices

Fifteen studies examined parental PTSD in relation to parenting styles (such as warmth, sensitivity, overprotection, hostility), parenting practices (e.g. reactive or inconsistent discipline), or parents’ potential to maltreat their child. Trauma types included intimate partner violence (Chemtob & Carlson, 2004; Schechter et al., 2010; Schechter et al., 2015), birth trauma (Davies et al., 2008; Forcada-Guex et al., 2011; Ionio & Di Blasio, 2014), military trauma (Gewirtz et al., 2010; Maršanić et al., 2015; Solomon, Debby-Aharon, Zerach, & Horesh, 2011; Vuković et al., 2015), learning of your child’s maltreatment (Jobe-Shields, Swiecicki, Fritz, Stinnette, & Hanson, 2016), and mixed traumas (Chemtob et al., 2013; Cross et al., 2017; Hershkowitz et al., 2017; Leen-Feldner et al., 2011). Overall, the quality ratings of papers were moderate to high (range .56–.87).^2^

#### 3.5.1. Studies using parental self-report

Nine studies examined self-reported discipline practices, measured via interview or questionnaire. The most consistently used measure was the Alabama Parenting Questionnaire (APQ), which includes domains of positive parenting, inconsistent discipline, and poor supervision (Elgar, Waschbusch, Dadds, & Sigvaldason, 2007; Frick, 1991). In the aforementioned population study of parents who had experienced varied traumas (*N* = 200), Hershkowitz et al. (2017) found overall PTSD scores to be correlated with poorer parenting behaviour scores, based on a combined score from the APQ (*r =* −0.24, indicating a small effect size). Effects were maintained when depressive symptoms were included in the same model. In a second study using the APQ, caregivers who developed PTSD secondary to their child being sexually abused showed higher levels of inconsistent discipline practices than those without PTSD. Effects were independent of caregiver depression status, and were present both based on caregivers’ own report and on reports of their child (*n*^2^ = 0.11; medium effect; Jobe-Shields et al., 2016). However, for other parenting practices (positive involvement, supervision/monitoring, positive discipline, corporal punishment) no significant PTSD effects were observed. In a key longitudinal study of 468 national guard fathers, Gewirtz et al. (2010) demonstrated that an increase in PTSD symptoms from initial assessment to one-year follow up was a predictor of less optimal parenting behaviours on the APQ at one-year (SEM standardized beta = –.36). Basic correlations were also presented, and these suggested that effects were present for each PTSD symptom cluster and mainly related to negative (inconsistent discipline/poor supervision) versus positive parenting practices. In mapping symptom change onto parenting outcomes, this important study moves a step closer to demonstrating causal effects, but the authors note that baseline measurement of parenting would be have been ideal in this respect.

Three studies examined a range of parenting practices using measures other than the APQ. In a study examining dysfunctional discipline practices in mothers who had experienced IPV, findings indicated mothers with PTSD (*n =* 11) reported significantly greater use of overall dysfunctional discipline strategies (*d =* 0.95), and reactive discipline in particular (*d =* 1.15), compared to exposed mothers with no PTSD (*n =* 14) (Chemtob & Carlson, 2004). Estimated effect sizes were large (calculated by this first author based on data reported in the publication). However, no significant differences were found between mothers with and without PTSD on laxness (e.g. neglect of the child) and verbosity (e.g. use of verbal controls). In one of the larger studies identified, Solomon et al. (2011) measured a range of parenting practices in veteran fathers using a five-item self-report measure, with items indexing: use of physical or verbal violence; extent of father’s involvement; cooperation from both parents in raising the child; and ability to meet the physical and emotional needs of the child. A single, total score was calculated. Veteran fathers with PTSD (*n =* 123) rated their own parenting behaviour as significantly lower on this scale compared to those without PTSD (*n =* 350). The effect size reported was large (*d =* 0.86; calculated by the first author). Although the scale used in this study showed good internal consistency, the fact that it combined divergent domains limits interpretability.

Three studies focused on overtly negative parenting practices in the context of parental PTSD. In a large study of parents exposed to varied traumas (Leen-Feldner et al., 2011), those with PTSD (*n =* 286) were significantly more likely to endorse aggressive parenting practices than parents without PTSD (*n =* 3644). Specifically, 72.5% of parents with PTSD reported using moderately aggressive parenting practices (e.g. spanking, slapping, grabbing, or pushing), compared to 62.5% of parents without PTSD (Cramer’s φ = 0.07; indicating a small effect size); use of severely aggressive parenting practices (e.g. kicking, or hitting with fist) was reported by 4.4% of parents with PTSD versus 2.4% of parents without (φ = 0.22; small effect). The large sample was a particular strength of this study, but no potential confounds were considered in analyses and a trauma-exposed control group was not specified. Cross et al. (2017) examined the impact of PTSD from lifetime trauma exposure on child abuse potential in low-income mothers (*n =* 112), 97% of whom reported trauma exposure. Findings indicated a significant correlation between mother’s PTSD scores and scores from the Child Abuse Potential Index (CAPI; Milner, 1994), with a large effect size (*r =* 0.57). Effects were maintained controlling for extent of maternal trauma exposure (β = .48). Lastly, in the previously described community sample of mothers (*N =* 97), a proportion of whom had experienced traumas, Chemtob et al. (2013) found that mothers with comorbid depression and PTSD reported elevated levels of physically (η^2^ = 0.17) and psychologically abusive (η^2^ = 0.19) behaviours^3^ towards their children, indicating large effect sizes, compared to mothers with no psychopathology diagnosis, depression-only, and PTSD-only. No differences between groups were found regarding child neglect.

#### 3.5.2. Studies using child report of parenting

Only two studies asked children themselves to report on their parenting experiences, which has the advantage of limiting the potential for informant bias. In a sample of adolescents who had a veteran father, those whose father was diagnosed with PTSD (*n =* 122) perceived them as providing significantly less care (Phi = 0.37), and using significantly more affectionless control (Phi = 0.24), compared to those without a paternal PTSD diagnosis (*n =* 122; Marsanic et al., 2014). No significant differences were found between groups regarding affectionate constraint or neglectful rearing practices. In a second study by the same research group, Vukovic et al. (2015) also studied adolescents of veteran fathers. Adolescents of fathers with full PTSD (*n =* 108) rated their fathers as significantly more controlling compared to adolescents of fathers with partial PTSD (*n =* 108) and no PTSD (*n =* 108). Furthermore, paternal care was scored significantly lower in the full and partial PTSD group compared to the non-PTSD group. Effect sizes reported ranged from moderate to large (*n*^2^ = 0.068–0.195). Although these studies provide a consistent picture of less caring and more controlling paternal behaviour in association with PTSD, it should be noted that adolescents were recruited from a psychiatric clinic in each case and findings may not generalize to other samples. Importantly, adolescents also reported on maternal parenting, and their perceptions of maternal warmth and care were similarly related to paternal PTSD. This suggests that paternal PTSD associations with parenting may have an underlying cause other than direct symptom effects.

#### 3.5.3. Studies utilizing direct observations of parenting behaviour

Four studies also examined parenting via direct observation. Although observational assessments are considered the gold standard in the parenting field, studies using this approach tended to have modest sample sizes. The focus has been on infancy and childhood, with infancy studies exclusively examining birth-related distress. In their small longitudinal study (*N =* 19), Ionio et al. (2014) found that higher scores of maternal PTSD two-months post-birth were cross-sectionally correlated with parenting during the still face paradigm (two-minute blocks of normal play, followed by still face, then a ‘reunion’ episode; see Tronick et al., 1978). Although some associations were identified between PTSD and parenting during both play and reunion phases, only one of these was consistent across both (making more mouth sounds; play Adj*R*^2^ = 0.31; reunion Ajd*R*^2^ = 0.24), which was coded as negative behaviour suggesting intrusiveness. Moreover, only nine significant associations were found in total, from an estimated 72 correlations carried out. Given the small sample, evidence, and lack of consistent effects, this study provides little evidence of parental PTSS influences on parenting.

A second study compared mothers who had given birth to pre-term infants, who were classified as PTSD (*n =* 16) versus no PTSD (*n =* 31) based on a self-report questionnaire, and mothers of full-term infants (*n =* 25) (Forcada-Guex et al., 2011). During a 10-minute dyadic interaction at infant age six months, pre-term mother–infant dyads in the maternal PTSD group were more likely to be classed as controlling mother–compliant infant than no PTSD or full-term groups. In addition, pre-term dyads were less likely to be classed as sensitive mother–cooperative infant than full-term dyads, regardless of PTSD status, whereas groups did not differ on the frequency of ‘heterogeneous’ interaction patterns (Forcada-Guex et al., 2011). Although suggestive, findings are difficult to interpret as parenting style was combined post-hoc with infant responding in all analyses. A further limitation was reliance on retrospective parental reporting of perinatal symptoms at 18-months postpartum.

Two observational studies examined IPV samples. In one of the larger observational studies, mothers exposed to IPV (*n =* 17 meeting diagnostic criteria for PTSD; *n =* 30 with sub-threshold symptoms; *n =* 27 with no PTSD symptoms) were observed during an interaction with their child aged 12 to 48 months (Schechter et al., 2010). Coding of ‘atypical’ maternal behaviours (e.g. withdrawal, lack of affective communication, and hostility) found no significant differences between groups. Nonetheless, exploratory post-hoc analyses found that maternal PTSD severity predicted the amount of time the child attempted unsuccessfully to engage the mother in joint attention after (but not before) a separation episode (β = 0.38, *p* < .001). This finding must be considered in the context of the wider set of null results from this study. In a more recent study by the same group, utilizing a similar method/sample, Schechter et al. (2015) found during a five-minute free play session with their child, mothers with IPV-PTSD or subthreshold IPV (*n =* 34) were significantly less sensitive, and significantly more controlling, compared to mothers who were IPV exposed but did not have PTSD (*n =* 22). Effect sizes were moderate to large (controlling *r =* 0.42; sensitivity *r =* −0.51, respectively). This study used a well-validated parenting index, which was a strength. However, there were some limitations, including the inclusion of subthreshold cases in the PTSD group and associated lack of information about the origin of cut-offs applied to define this group, and the identification of differences in extent of trauma exposure in PTSD versus no PTSD groups that were not controlled for in analyses.

Overall, it is apparent that there is a mix of methodological approaches to investigating the impact of parental PTSD on specific parenting practices. While results generally suggest that parental PTSD is associated with use of more negative parenting practices, studies still have a heavy reliance on parental self-report, which may not provide an accurate representation. However, as the most investigated area, results in relation to parenting practices provide evidence across multiple trauma types, which is positive when generalizing to other trauma exposed populations.

## 4. Discussion

The available evidence suggests that parental PTSD is associated with elevated levels of parenting stress, as well as being associated with detrimental effects to parenting satisfaction, the parent–child relationship, and the endorsement of negative parenting practices. Such effects are reported relatively consistently, albeit with substantial variability in terms of what is indexed under each of these constructs. At the same time, there are some limitations to the field, which mean that it would be premature to draw firm conclusions.

Studies reviewed provided relatively consistent evidence that parental PTSD is associated with reduced parenting satisfaction, albeit with some contradictory findings. Effect sizes, as well as sample sizes, were generally small, and positive findings derived predominantly from studies of male military veterans – further evidence is needed to establish their generalizability. While there is clearly potential for reduced parenting satisfaction to result in actual impairments in the parent–child relationship, one study to address this failed to identify a pathway from satisfaction to parenting behaviour (Hershkowitz et al., 2017), and this question was generally underexplored. Even if direct implications for parental behaviour and/or child outcomes are not established, poor parental satisfaction seems likely to compound parental distress, which is important in clinical terms (Sherman, Larsen, Starits-Troster, Erbes, & Tassey, 2015). The consequences of reduced parenting satisfaction in the context of PTSD warrant further examination.

While only a small number of studies included investigated the impact of parental PTSD on parenting stress, the results were generally consistent in suggesting parental PTSD is associated with increased parenting stress. The use of the same measure across all four studies is a strength, as is the inclusion of heterogenous trauma samples. However, three of the four studies only reported a total score of the PSI-SF. In order to further unpack and understand the influence each of the measured sub-scales (e.g. perceived difficulty of the child parental distress or parent–child dysfunction) has on parenting stress, future studies should seek to include each of the sub-scale totals in their analysis, as well as the total composite score.

Parental perceptions have also been studied in relation to the parent–child relationship, with studies in this area particularly focusing on PTSD associated with birth experiences. Findings have been mixed: of three studies measuring parents’ self-reported bond to their infant, only one provided reliable evidence of an association with PTSD symptoms per se (versus, for example, trauma exposure). Two studies that examined parents’ attachment to their infant found little robust evidence of an association with parental PTSD. Finally, one study examined infant attachment to their mother using direct observation in the strange situation and found lower rates of secure attachments, and particularly elevated levels of disorganized infant attachments, in association with maternal PTSD. Effects in this study were substantial, and the study was notable in having an independent (versus parent-reported) indicator of parent–infant relationship quality. Nonetheless, conclusions were based on only 45 participants, 12 with PTSD. As such, although evidence of attachment insecurity in association with parental PTSD is of real concern, given potential links with problematic parenting behaviours and long-term adverse child outcomes across a range of domains, replication of these observations is critically needed.

A substantial proportion of studies in the current review focused on aspects of parenting behaviour, using samples that encompassed a range of trauma types and child ages. Studies based on parental self-report provided relatively consistent evidence that parental PTSD is associated with more negative parenting, including inconsistent/reactive discipline, controlling behaviours, and displays of overt hostility and aggression. By contrast, positive parenting strategies were not linked to parental PTSD in the subset of studies that examined those, suggesting that associations do not simply reflect more negative parental self-perceptions overall. A recent review of parenting following child maltreatment highlighted that some first-time parents report positive associations between their trauma and their parenting (Christie et al., 2018; Fava et al., 2016). Further, in a recent qualitative study with parents in a South African township, parents were still able to find positive aspects in their relationship with their child despite experiences of trauma (Christie et al., in prep). This may suggest that positive elements of parenting may be conserved even in the context of parental PTSD, which warrants further investigation.

The majority of studies focused on deviations in more normative parenting practices (e.g. inconsistent/reactive discipline or intrusive/controlling behaviours), which nonetheless may increase children’s risk for developing both internalizing and externalizing disorders (Padillia-Walker, 2008). However, there were also observations that parental PTSD may result in hostile or more severely aggressive parenting practices, which are of particular concern, albeit with small effect. Importantly, one longitudinal study also provided evidence that *change* in parental PTSD symptoms predicted subsequent parenting behaviours, consistent with a possible causal role of posttraumatic distress. However, parenting was not measured at baseline in this study and other explanations (e.g. the presence of underlying and persistent parenting or family environment problems that exacerbate parental PTSD) could not be ruled out.

By contrast to the evidence based on parents’ own reports, studies that used direct observations or child reports of parenting behaviours provided less reliable evidence. Of four studies that used observational assessments with mother–infant dyads, only one found clear evidence of less optimal parenting in association with maternal PTSD (Schechter et al., 2015). Two studies using child/adolescent informants to measure parenting both provided evidence of less optimal parenting in association with parental PTSD. However, these studies found that adolescents who had a veteran father with PTSD reported more negative parenting for both their fathers and their mothers than a comparison group without paternal PTSD. Of course, there could be many reasons why paternal PTSD has a knock-on impact on maternal parenting, but such observations call into question the assumed direct causal impact of PTSD symptoms on parental behaviour. Moreover, studies using child informants had samples of young people selected based on specific characteristics, namely the presence of maltreatment or mental health problems, so findings may not generalize.

Overall, whilst it seems clear that parents with PTSD perceive themselves to be worse parents and obtain less satisfaction from their parenting role, the extent to which this reflects actual parenting impairments is less clear. The reliance on cross-sectional studies in the field is striking, and causal evidence to demonstrate that parental PTSD is having a direct impact on parenting domains is particularly lacking. The possibility that pre-existing or co-occurring risk factors (e.g. poor family environment, substance use) explain associations often cannot be ruled out. The use of rigorous longitudinal methodology to examine temporal influences between parental PTSD and parenting domains over time would provide better evidence of causal influence. Similarly, measurement of parenting in the context of intervention studies could provide powerful evidence of direct causal influences of posttraumatic distress. Both of these approaches will require larger samples than many of those reported in the current review.

Within cross-sectional designs, taking a more rigorous approach to measuring and controlling for key potential confounds is also critical to strengthen the evidence base. First, with some notable exceptions (Bosquet Enlow et al., 2014; Davies et al., 2008), there was limited attention to trauma type and/or symptom severity in the studies reviewed. For example, studies may not have explicitly recruited a Criterion A trauma exposed sample. In cases where a Criterion A trauma sample had been recruited, it was noted that some participants no longer experienced any distress or traumatic symptoms from their trauma exposure, yet this was not taken into account during the analysis. Further, variations in the nature of the trauma may directly impact parental domains (e.g. studies of premature births). The fact that some studies found evidence that trauma per se may be associated with altered parental functioning suggests that the literature as a whole should attempt to take account of key trauma characteristics.

Second, some studies looked at co-occurring mental health problems in parents, particularly parental depression, with mixed conclusions as to whether PTSD effects may be secondary to other forms of psychological disorders (e.g. Davies et al., 2008; Samper et al., 2004). Although disentangling impacts of co-occurring disorders is likely to be challenging, more consistent measurement and analysis in key areas (especially depression and alcohol and substance abuse) is necessary to provide a reliable picture of potential PTSD related impacts.

Third, not all studies reported on or controlled for key demographic characteristics linked to mental health and parenting behaviour (e.g. level of education). More rigorous consideration of potential confounds will considerably strengthen the case for a direct causal influence of parental PTSD on parenting. Finally, making use of multiple informants and/or independent observations of parenting is crucial to rule out inflation of effects by informant bias. This is especially relevant when measuring the parent–child relationship, as PTSD has been associated with subjective sense of relationship dysfunction which could influence self-report (Schechter et al., 2015).

Although the aim of the current review was to consider *whether* parental PTSD has an impact on parenting, it will also be important to learn more about *why* such impacts are present. In this respect, some of the reviewed studies examined specific PTSD symptom clusters, and there was some evidence that avoidance or hyperarousal may be more strongly related to parenting than intrusive symptoms (Berz et al., 2008; Samper et al., 2004). This is consistent with qualitative evidence in which feelings of irritability/anger and a need to avoid trauma reminders were viewed as particularly problematic by trauma exposed parents (Christie et al., in prep; Sherman et al., 2015), but requires more examination. Such work may ultimately provide better information about whether and how interventions for PTSD are simultaneously likely to deliver improvements in parenting domains.

Some further considerations should be taken into account in interpreting the current findings. Due to the fundamental complexity of parenting as a construct, there was significant variation across studies with regards to focal domains, and there were associated inconsistencies in terms of the precise nature and magnitude of effects reported. Many studies had modest sample sizes, which also limits potential to obtain precise estimates of effect sizes. The quality ratings of some of the included studies were classed as low to moderate due to issues with the conduct and reporting of the research. Certain types of trauma (particularly birth and military trauma) are disproportionately represented in the literature, which limits generalizability, and fathers are under-represented in parental PTSD research. Future research should seek to address these issues.

## 5. Conclusion

Parental PTSD may have a negative impact on parenting domains, but it is not clear whether effects are equally likely to apply across all trauma populations, and causal evidence is extremely limited. In addition, some methodological limitations in the extant literature need to be addressed. Nonetheless, clinicians should be conscious of the potential for PTSD to have an impact on parents and their parenting domains. An awareness of these negative impacts could give rise to more tailored support being provided for families following a parent’s trauma exposure.

## Supplementary Material

Supplemental Material

## Notes

1. A full reference list of all studies included in the review can be found in Supplemental data.

2. A subset of findings from studies have been reported in previous sections (Chemtob et al., 2013; Forcada-Guex et al., 2011; Hershkowitz et al., 2017).

3. As measured by the parent-to-child version of the Conflict Tactics Scale (CTS-PC).
