# Supplementary material for: The impact of parental posttraumatic stress disorder on parenting: a systematic review
Source: Eur J Psychotraumatol. 2019 Jan 14;10(1):1550345. doi: 10.1080/20008198.2018.1550345 (PMC6338266; doi:10.1080/20008198.2018.1550345)
Supplement: Supplemental Material [file ZEPT_A_1550345_SM3511.docx]

**Supplementary Material**

**References (of those included in the systematic review)**

Ayers, S., Wright, D. B., & Wells, N. (2007). Symptoms of post-traumatic stress disorder in couples after birth: Association with the couple's relationship and parent-baby bond. *Journal of Reproductive and Infant Psychology, 25*(1), 40-50.

Berz, J. B., Taft, C. T., Watkins, L. E., & Monson, C. M. (2008). Associations between PTSD symptoms and parenting satisfaction in a female veteran sample. *Journal of Psychological Trauma, 7*(1), 37-45.

Bosquet Enlow, M., Egeland, B., Carlson, E., Blood, E., & Wright, R. J. (2014). Mother-infant attachment and the intergenerational transmission of posttraumatic stress disorder. *Developmental Psychopathology, 26*(1), 41-65.

Chemtob, C. M., & Carlson, J. G. (2004). Psychological effects of domestic violence on children and their mothers. *International Journal of Stress Management, 11*(3), 209-226.

Chemtob, C. M., Gudiño, O. G., & Laraque, D. (2013). Maternal Posttraumatic Stress Disorder and Depression in Pediatric Primary Care Association with Child Maltreatment and Frequency of Child Exposure to Traumatic Events. *JAMA Pediatrics, 167*(11), 1011-1018.

Cohen, E., Zerach, G., & Solomon, Z. (2011). The implication of combat-induced stress reaction, PTSD, and attachment in parenting among war veterans. *Journal of Family Psychology, 25*(5), 688-698.

Creech, S. K., Swift, R., Zlotnick, C., Taft, C., & Street, A. E. (2016). Combat exposure, mental health, and relationship functioning among women veterans of the Afghanistan and Iraq wars. *Journal of Family Psychology*, *30*(1), 43-51.

Cross, D., Vance, L. A., Kim, Y. J., Ruchard, A. L., Fox, N., Jovanovic, T., & Bradley, B. (2017). Trauma exposure, ptsd, and parenting in a community sample of low-income, predominantly african american mothers and children. *Psychological Trauma: Theory, Research, Practice, and Policy*, 10(3), 327-335.

Davies, J., Slade, P., Wright, I., & Stewart, P. (2008). Posttraumatic stress symptoms following childbirth and mothers' perceptions of their infants. *Infant Mental Health Journal, 29*(6), 537-554.

Forcada-Guex, M., Borghini, A., Pierrehumbert, B., Ansermet, F., & Muller-Nix, C. (2011). Prematurity, maternal posttraumatic stress and consequences on the mother–infant relationship. *Early Human Development, 87*(1), 21-26.

Gewirtz, A. H., Polusny, M. A., DeGarmo, D. S., Khaylis, A., & Erbes, C. R. (2010). Posttraumatic stress symptoms among National Guard soldiers deployed to Iraq: Associations with parenting behaviors and couple adjustment. *Journal of Consulting and Clinical Psychology, 78*(5), 599-610.

Hershkowitz, M., Dekel, R., Fridkin, S., & Freedman, S. (2017). Posttraumatic stress disorder, parenting, and marital adjustment among a civilian population. Frontiers *in Psychology, 4*(8), 1655.

Ionio, C., & Di Blasio, P. (2014). Post-traumatic stress symptoms after childbirth and early mother–child interactions: An exploratory study. *Journal of Reproductive and Infant Psychology, 32*(2), 163-181.

Jobe-Shields, L., Swiecicki, C. C., Fritz, D. R., Stinnette, J. S., & Hanson, R. F. (2016). Posttraumatic stress and depression in the nonoffending caregivers of sexually abused children: Associations with parenting practices. *Journal of Child Sexual Abuse: Research, Treatment, & Program Innovations for Victims, Survivors, & Offenders, 25*(1), 110-125.

Jordan, B. K., Marmar, C. R., Fairbank, J. A., Schlenger, W. E., Kulka, R. A., Hough, R. L., & Weiss, D. S. (1992). Problems in families of male Vietnam veterans with posttraumatic stress disorder. *Journal of Consulting and Clinical Psychology, 60*(6), 916-926.

Lauterbach, D., Bak, C., Reiland, S., Mason, S., Lute, M. R., & Earls, L. (2007). Quality of parental relationships among persons with a lifetime history of posttraumatic stress disorder. *Journal of Traumatic Stress, 20*(2), 161-172.

Leen-Feldner, E. W., Feldner, M. T., Bunaciu, L., & Blumenthal, H. (2011). Associations between parental posttraumatic stress disorder and both offspring internalizing problems and parental aggression within the National Comorbidity Survey-Replication. *Journal of Anxiety Disorders, 25*(2), 169-175.

Maršanić, V. B., Margetić, B. A., Bulić, S. O., Đuretić, I., Kniewald, H., Jukić, T., & Paradžik, L. (2015). Non-suicidal self-injury among psychiatric outpatient adolescent offspring of Croatian posttraumatic stress disorder male war veterans: Prevalence and psychosocial correlates. *International Journal of Social Psychiatry, 61*(3), 265-274.

Parfitt, Y. M., & Ayers, S. (2009). The effect of post-natal symptoms of post-traumatic stress and depression on the couple's relationship and parent-baby bond. *Journal of Reproductive and Infant Psychology, 27*(2), 127-142.

Salloum, A., Stover, S. C., Swaidan, V. R., & Storch, E. A. (2015). Parent and Child PTSD and Parent Depression in Relation to Parenting Stress Among Trauma-Exposed Children. *Journal of Child and Family Studies, 24*(5), 1203-1212.

Samper, R. E., Taft, C. T., King, D., & King, L. A. (2004). Posttraumatic Stress Disorder Symptoms and parenting satisfaction among a national sample of male Vietnam weterans. *Journal of Traumatic Stress, 17*(4), 311 - 315.

Schechter, D. S., Suardi, F., Manini, A., Cordero, M. I., Rossignol, A. S., Merminod, G., Gex-Fabry, M., Moser, D. A., & Serpa, S. R. (2015). How do maternal PTSD and alexithymia interact to impact maternal behavior? *Child Psychiatry and Human Development, 46*(3), 406-417.

Schechter, D. S., Willheim, E., Hinojosa, C., Scholfield-Kleinman, K., Turner, J. B., McCaw, J., Zeanah, C. H., & Myers, M. M. (2010). Subjective and objective measures of parent-child relationship dysfunction, child separation distress, and joint attention. *Psychiatry: Interpersonal and Biological Processes, 73*(2), 130-144.

Solomon, Z., Debby-Aharon, S., Zerach, G., & Horesh, D. (2011). Marital adjustment, parental functioning, and emotional sharing in war veterans. *Journal of Family Issues, 32*(1), 127-147.

Suttora, C., Spinelli, M., & Monzani, D. (2014). From prematurity to parenting stress: The mediating role of perinatal post-traumatic stress disorder. *European Journal of Developmental Psychology, 11*(4), 478-493.

Vuković, I. S., Maršanić, V. B., Margetić, B. A., Paradžik, L., Vidović, D., & Flander, G. B. (2015). Self-reported emotional and behavioral problems, family functioning and parental bonding among psychiatric outpatient adolescent offspring of Croatian male veterans with partial PTSD. *Child Youth Care Forum, 44*(5), 655-669.

Wilson, C. K., Padrón, E., & Samuelson, K. W. (2017). Trauma type and posttraumatic stress disorder as predictors of parenting stress in trauma-exposed mothers. *Violence and Victims, 32*(1), 141-158.
